# Identification of key genes in lung adenocarcinoma based on a competing endogenous RNA network

**DOI:** 10.3892/ol.2020.12322

**Published:** 2020-11-19

**Authors:** Zikun Song, Yinjiang Zhang, Zheren Chen, Bicheng Zhang

**Affiliations:** 1 Department of Intensive Care Medicine, The People's Second Hospital of Liaocheng, Linqing, Shandong 252601, P.R. China; 2 School of Pharmacy, Minzu University of China, Beijing 100081, P.R. China; 3 Department of Oncology, Renmin Hospital of Shishou, Jingzhou, Hubei 434400, P.R. China; 4 Department of Oncology, Renmin Hospital of Wuhan University, Wuhan, Hubei 430070, P.R. China

**Keywords:** receptor activity modifying protein 2-antisense RNA 1, microRNA-296-5p, competing endogenous RNA, long non-coding RNA, lung adenocarcinoma

## Abstract

Lung adenocarcinoma (LUAD) is the most commonly diagnosed type of lung cancer and exhibits a high morbidity. The present study aimed to investigate the long non-coding RNA (lncRNA)-associated competing endogenous RNA (ceRNA) mechanisms in LUAD. The receptor activity modifying protein 2-antisense RNA 1 (RAMP2-AS1) was identified using GSE113852 and GSE130779 datasets downloaded from the Gene Expression Omnibus database, and the downregulation of RAMP2-AS1 was the most significant in LUAD. In addition, microRNA (miR)-296-5p was identified to bind to RAMP2-AS1 via bioinformatics analysis. Subsequently, CD44, cyclin D3 (CCND3), neurocalcin δ (NCALD), microtubule actin crosslinking factor 1 (MACF1) and potassium channel tetramerization domain containing 15 were obtained by intersecting the predicted target genes of miR-296-5p and 368 differentially expressed mRNAs in LUAD. According to the Gene Expression Profiling Interactive Analysis and UALCAN databases, these five mRNAs were downregulated in LUAD, and their expression levels were positively correlated with those of RAMP2-AS1. CD44, CCND3, NCALD and MACF1 were selected as key mRNAs in LUAD based on prognostic analyses. Furthermore, functional enrichment analyses were performed and an interaction network was constructed to reveal the functions of the RAMP2-AS1-associated ceRNA in LUAD. The results indicated that the functions were mainly enriched in generic transcription pathways, cyclin D-associated events in G_1_ and epithelial stromal transformation. Reverse transcription-quantitative PCR assays revealed that RAMP2-AS1, CD44, CCND3, NCALD and MACF1 expression was lower in tumor tissues than in normal tissues, while miR-296-5p expression was higher in tumor tissues compared with in normal tissues. The association between RAMP2-AS1 and MACF1 was further confirmed using *in vitro* experiments. Overall, the present results indicated that RAMP2-AS1, miR-296-5p, CD44, CCND3, NCALD and MACF1 may be involved in LUAD progression and may therefore serve as potential biomarkers and provide a theoretical basis for the study of the pathogenesis of LUAD.

## Introduction

Lung cancer is the leading cause of cancer-associated deaths worldwide (1), with the 5-year survival rate of patients with lung cancer being <20% (2). Non-small cell lung cancer accounts for >85% of lung cancer cases and ~60% of these cases are classified as lung adenocarcinoma (LUAD) (3). At the time of diagnosis, ~70% of patients with lung cancer have locally advanced or metastatic disease (4). Although the development of anti-angiogenic drugs, EGFR inhibitors and other novel anticancer agents has greatly improved the treatment of lung cancer, the 5-year survival rate remains <15% (5). Therefore, the present study aimed to provide a valuable theoretical basis for the study of the mechanisms underlying the development of LUAD and novel directions for the further investigation of the pathogenesis of LUAD.

It has been reported that competing endogenous RNAs (ceRNAs) serve an important role in the post-transcriptional regulation of genes by competing with other RNA molecules to bind to specific microRNAs (miRNAs/miRs) via common miRNA response elements (6,7). Numerous studies have demonstrated that the regulatory mechanisms of ceRNAs are critical in the development and progression of several types of cancer, including breast (8), bladder (9) and lung cancer (10). An increasing number of long non-coding RNAs (lncRNAs) have been identified to serve vital roles in the pathogenesis of LUAD through the mechanisms of ceRNAs (11,12). For example, Dong *et al* (13) demonstrated that the lncRNA DiGeorge syndrome critical region gene 5 promoted LUAD progression via inhibiting hsamiR-22-3p. Additionally, Xiong *et al* (14) revealed that lncRNA nuclear paraspeckle assembly transcript 1 (NEAT1) accelerated LUAD deterioration by acting as a ceRNA to regulate miR-193a-3p expression. Furthermore, it has been reported that exosomes derived from chondrosarcoma cells carry the lncRNA receptor activity modifying protein 2-antisense RNA 1 (RAMP2-AS1), which acts as a ceRNA of miR-2355-5p to modulate the expression levels of vascular endothelial growth factor receptor 2 (VEGFR2), thus actively regulating the angiogenic ability of human umbilical vein endothelial cells (HUVECs) (15). Consequently, it has been speculated that exosomes carrying RAMP2-AS1 may be a novel biomarker and therapeutic target for chondrosarcoma (15). Therefore, exploring lncRNA-associated ceRNA mechanisms in LUAD may lead to the development of effective diagnostic and therapeutic strategies.

Over the past few decades, with the development of high-throughput technology, rapid progress has been made in the identification of differentially expressed genes to further explore the molecular mechanisms underlying cancer development. In the present study, two microarray datasets (GSE113852 and GSE130779) were downloaded from the Gene Expression Omnibus (GEO) to identify the key lncRNAs and mRNAs. Furthermore, a functional enrichment analysis was performed and an interaction network was constructed to explore the functions of the key genes associated with LUAD. The present study aimed to identify the key genes associated with the development of LUAD and to provide available target genes for the treatment and diagnosis of LUAD by performing bioinformatics analyses.

## Materials and methods

### 

#### Identification of key lncRNAs and mRNAs

GSE113852 and GSE130779 gene expression profiles were downloaded from the GEO database (http://www.ncbi.nlm.nih.gov/geo/). In the GSE113852 dataset, GSM3121285-GSM3121311 and GSM3121312-GSM3121338 included 27 paired normal lung and lung cancer samples, respectively. In the GSE130779 dataset, GSM3753429-GSM3753432 and GSM3753437-GSM3753440 included 8 LUAD samples, whereas GSM3753433-GSM3753436 and GSM3753441-GSM37534344 included 8 normal paired samples. Subsequently, differential gene expression analysis was performed using the GEO2R (http://www.ncbi.nlm.nih.gov/geo/geo2r/) web tool with a threshold of |log2 fold-change (FC)| >1 and P<0.05. Subsequently, the differentially expressed lncRNAs and mRNAs in the two datasets were identified using Venn diagrams (Venny2.1https://bioinfogp.cnb.csic.es/tools/venny/index.html). The gene expression profile of lung adenocarcinoma was obtained from The Cancer Genome Atlas (TCGA) database (https://tcga-data.nci.nih.gov/tcga/). The Starbase V2.0 (http://starbase.sysu.edu.cn/starbase2/) database was used to screen for miRNAs that could bind to RAMP2-AS1 as ceRNAs. Starbase V2.0 is a database used for the systematical identification of RNA-RNA and protein-RNA interaction networks (16). Furthermore, the target genes of miR-296-5p were obtained via TargetScan (17) (http://www.targetscan.org/vert_72/) and miRDB (18) (http://mirdb.org/) databases. Both these online databases are used for miRNA target prediction and functional annotations. The expression pattern of miR-296-5p in LUAD was acquired from the database of differentially expressed miRNAs in human Cancers (dbDEMC; (http://www.picb.ac.cn/dbDEMC/index.html) (19). Finally, the target genes were intersected with differentially expressed mRNAs to obtain the key mRNAs.

#### Expression and prognostic analyses

The expression status of RAMP2-AS1 in tumors was acquired using the Gene Expression Profiling Interactive Analysis (GEPIA; http://gepia.cancer-pku.cn/detail.php) online tool. GEPIA is a web server for gene expression profiling and interactive analyses of normal and cancer samples (20). In addition, the UALCAN platform (http://ualcan.path.uab.edu/) is an interactive web-portal for facilitating tumor subgroup gene expression and survival analyses (21). Therefore, this platform was used to investigate the expression levels of key mRNAs in LUAD and adjacent normal tissues, as well as their association with cancer stage (22), nodal metastasis status (23) and histological subtype. N0 represents no regional lymph node metastasis; N1 represents metastases in 1–3 axillary lymph nodes; N2 represents metastases in 4–9 axillary lymph nodes; N3 represents metastases in ≥10 axillary lymph nodes. The correlation between the expression of RAMP2-AS1 and the key mRNAs in LUAD was analyzed using Spearman's rank correlation test in GEPIA. The overall survival (OS) analysis of RAMP2-AS1 was also evaluated using the GEPIA online tool. Finally, the OS and first progression (FP) analyses of key mRNAs were performed using Kaplan-Meier plotter (24) (2014 version). The log-rank test was used to determine differences in the survival rate between the high and low expression groups.

#### LUAD samples

Tumor and adjacent normal tissue samples (>2 cm from tumor) used in the present study were collected from surgery from 40 patients with LUAD at Zhongnan Hospital of Wuhan University (Wuhan, China) between January 2018 and January 2019. The patients included 22 males and 18 females, and had a median age of 64 years (range, 37–75 years). None of the patients had received any anticancer therapy prior to surgery. Each patient provided written informed consent, which was in accordance with the ethical guidelines of Zhongnan Hospital of Wuhan University. Additionally, the collection of human tumor tissues was approved by the Ethical Committee of Zhongnan Hospital of Wuhan University.

#### Functional enrichment analysis

The cBio Cancer Genomics Portal (cBioPortal; http://cbioportal.org) was used to investigate the interactions between the key mRNAs and obtain the important genes involved. cBioPortal provides a web resource for exploring, visualizing and analyzing multidimensional cancer genomics data, enabling researchers to interactively explore genetic alterations across samples, genes and pathways (25). Furthermore, Gene Ontology (GO) and Kyoto Encyclopedia of Genes and Genomes (KEGG) pathway function enrichment analyses were performed using the FunRich software (version 3.1.3) (26). This open access functional enrichment and network analysis tool provides graphical representation, such as Venn, pie charts and heatmaps, of the data with customizable font, scale and color (26).

#### Interaction network construction

The Search Tool for the Retrieval of Interacting Genes/Proteins (STRING; http://string-db.org/) database incorporates known and predicted protein-protein association data for a large number of organisms, including direct (physical), as well as indirect (functional) interactions (27). Therefore, based on the STRING database, the ceRNA network was constructed using Cytoscape (version 3.6.1) (28), which is an open source software project that integrates biomolecular interaction networks with expression profiles, phenotypes and other molecular states into a unified conceptual framework.

#### Reverse transcription-quantitative PCR (RT-qPCR) assays

Total RNA was extracted from LUAD tissues using TRIzol^®^ reagent (Invitrogen; Thermo Fisher Scientific, Inc.), according to the manufacturer's protocol. Following total RNA isolation, RT was performed according to the protocol of the UEIris II RT-PCR System for First-Strand cDNA Synthesis (US Everbright^®^ Inc.) kit. The SYBR Premix Ex Taq (US Everbright^®^ Inc.) kit was employed to perform qPCR (denaturation, 30 sec at 95°C; annealing, 30 sec at 58°C; extension, 30 sec at 72°C; 35 cycles) on the ABI 7900 system (Applied Biosystems; Thermo Fisher Scientific, Inc.) and GAPDH served as the endogenous control. Comparative quantification was performed using the 2^−ΔΔCq^ method (29). The primers were purchased from Sangon Biotech Co., Ltd., and their sequences are listed in Table I.

#### Statistical analysis

Statistical analyses were performed using GraphPad Prism (version 7.0; GraphPad Software, Inc.). Data are expressed as the mean ± standard deviation. Comparisons between two groups (normal vs. tumor tissues) were analyzed using a paired Student's t-test. Comparisons among multiple groups were analyzed using one-way ANOVA followed by Tukey's post hoc test. All experiments were performed in triplicate. P<0.05 was considered to indicate a statistically significant difference.

## Results

### 

#### Identification of key lncRNA and mRNAs

**Figure 1.**
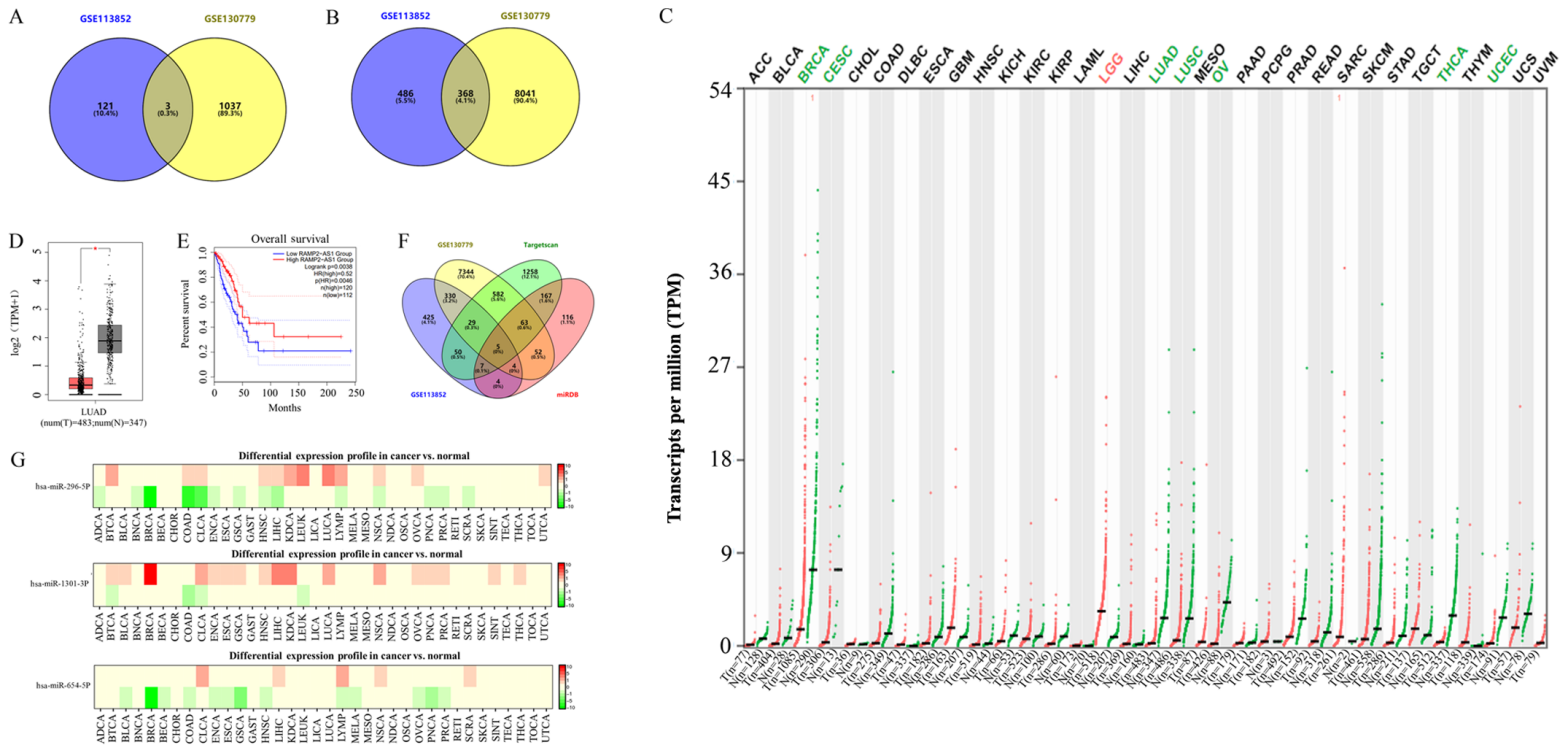
Identification of key lncRNAs and mRNAs in LUAD. Venn diagrams of the intersection of differentially expressed (A) lncRNAs and (B) mRNAs from the GSE113852 and GSE130779 datasets. (C) Expression levels of RAMP2-AS1 in different types of cancer as analyzed using TCGA. Green indicates that RAMP2-AS1 expression was downregulated, while red indicates that it was upregulated. (D) Expression levels of RAMP2-AS1 in LUAD and normal tissues as analyzed using TCGA. (E) Prognostic analysis of RAMP2-AS1 expression using Gene Expression Profiling Interactive Analysis. (F) Venn diagrams of the intersection of 368 differentially expressed mRNAs and the target genes of miR-296-5p identified using TargetScan and miRDB. (G) Differential expression profile of miR-296-5p, miR-1301-3P and miR-654-5P. Green indicates downregulation, while red indicates upregulation. T, tumor; N, normal; lncRNA, long non-coding RNA; LUAD, lung adenocarcinoma; RAMP2-AS1, receptor activity modifying protein 2-antisense RNA 1; TCGA, The Cancer Genome Atlas; ACC, adrenocortical carcinoma; BLCA, bladder urothelial carcinoma; BRCA, breast invasive carcinoma; CESC, cervical squamous cell carcinoma and endocervical adenocarcinoma; CHOL, cholangiocarcinoma; COAD, colon adenocarcinoma; DLBC, lymphoid neoplasm diffuse large B-cell lymphoma; ESCA, esophageal carcinoma; GBM, glioblastoma multiforme; HNSC, head and neck squamous cell carcinoma; KICH, kidney chromophobe; KIRC, kidney renal clear cell carcinoma; KIRP, kidney renal papillary cell carcinoma; LAML, acute myeloid leukemia; LGG, brain lower grade glioma; LIHC, liver hepatocellular carcinoma; LUSC, lung squamous cell carcinoma; MESO, mesothelioma; OV, ovarian serous cystadenocarcinoma; PAAD, pancreatic adenocarcinoma; PCPG, pheochromocytoma and paraganglioma; PRAD, prostate adenocarcinoma; READ, rectum adenocarcinoma; SARC, sarcoma; SKCM, skin cutaneous melanoma; STAD, stomach adenocarcinoma; TGCT, testicular germ cell tumors; THCA, thyroid carcinoma; THYM, thymoma; UCEC, uterine corpus endometrial carcinoma; UCS, uterine carcinosarcoma; UVM, uveal melanoma; TPM, transcripts per million.

A total of three differentially expressed lncRNAs (Fig. 1A) and 368 differentially expressed mRNAs (Fig. 1B) were obtained from the GSE113852 and GSE130779 datasets using the GEO2R and Venn diagrams. Among them, lncRNA RAMP2-AS1 was the most significantly downregulated (Table II) and was therefore chosen for subsequent studies. Samples were divided according to the expression levels of all analyzed RNAs from high to low, with values higher than the median value considered as high expression and values lower than the median value considered as low expression. TCGA results revealed that RAMP2-AS1 expression was downregulated in the majority of tumor types (Fig. 1C) and was significantly downregulated in LUAD tissues compared with in normal tissues (Fig. 1D). Furthermore, the prognostic analysis in GEPIA revealed that increased expression levels of RAMP2-AS1 were associated with an improved OS (Fig. 1E). Furthermore, three miRNAs binding to RAMP2-AS1 were identified using Starbase, namely miR-296-5p, miR-1301-3p and miR-654-5p. However, according to the dbDEMC, the differential expression of miR-296-5p was the most marked in LUAD (Fig. 1G). Subsequently, the target genes of miR-296-5p were obtained using TargetScan and miRDB databases. The intersection of these target genes with the 368 differentially expressed mRNAs identified in the aforementioned datasets revealed five key mRNAs, namely CD44, cyclin D3 (CCND3), neurocalcin δ (NCALD), microtubule actin crosslinking factor 1 (MACF1) and potassium channel tetramerization domain containing 15 (KCTD15) (Fig. 1F).

#### Expression and prognostic analyses

**Figure 2.**
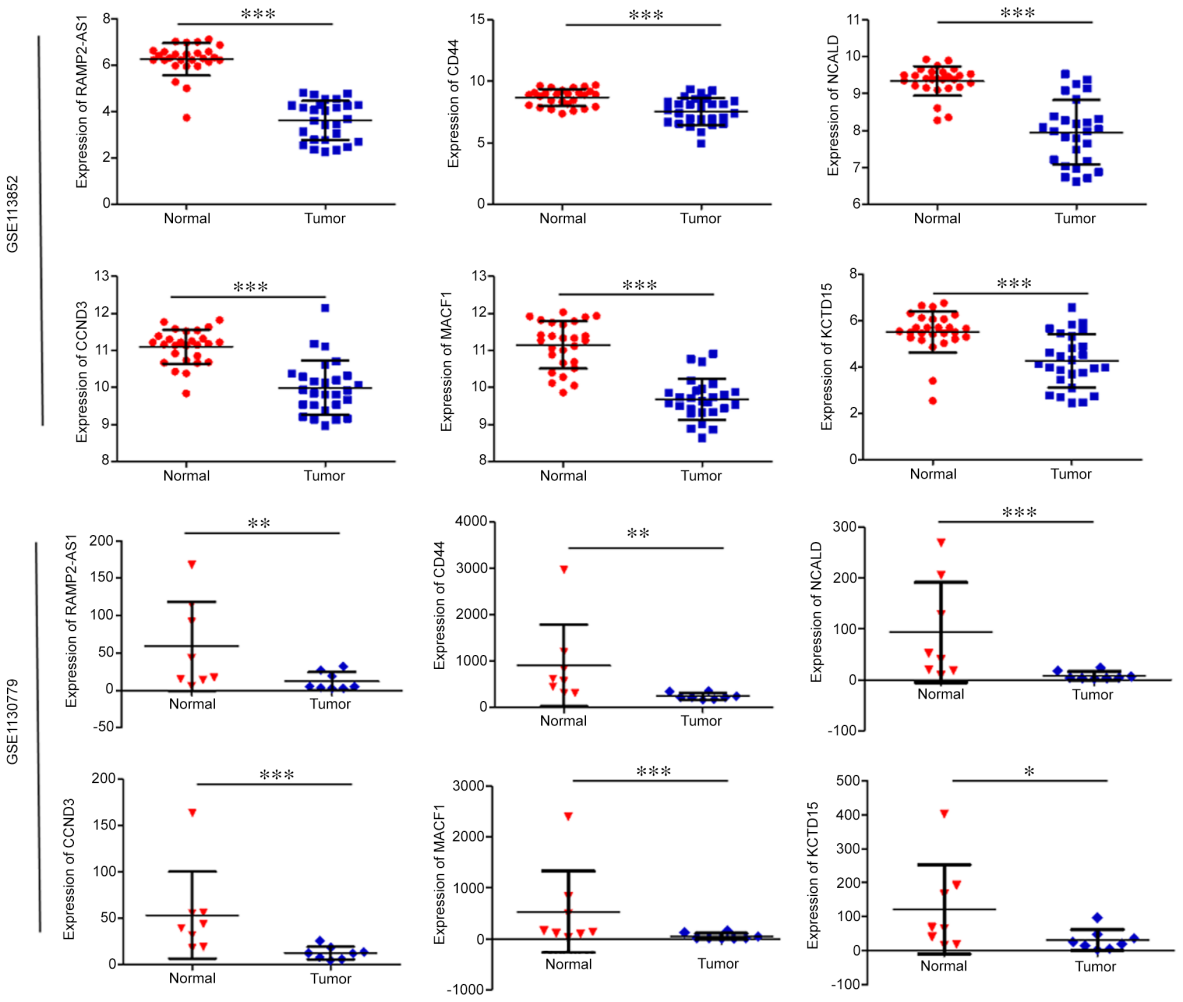
Expression levels of the selected genes in lung adenocarcinoma and adjacent normal tissues derived from the GSE113852 and GSE130779 datasets. *P<0.05; **P<0.01; ***P<0.001. CCND3, cyclin D3; NCALD, neurocalcin δ; MACF1, microtubule actin crosslinking factor 1; RAMP2-AS1, receptor activity modifying protein 2-antisense RNA 1; KCTD15, potassium channel tetramerization domain containing 15.

**Figure 3.**
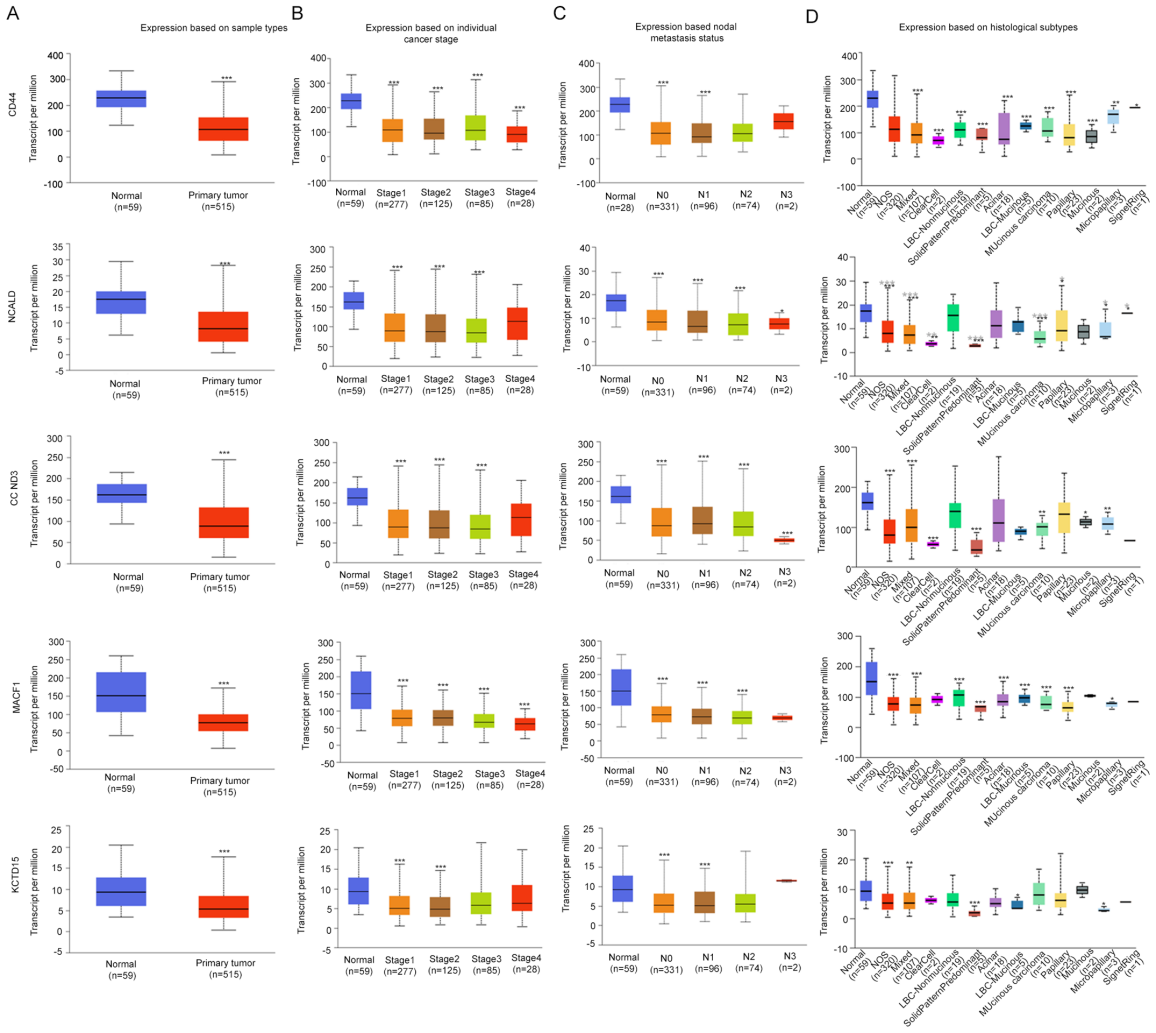
Expression levels of the selected genes in the UALCAN database. Expression levels of selected genes in lung adenocarcinoma based on (A) sample type, (B) individual cancer stage, (C) nodal metastasis status and (D) histological subtype. *P<0.05; **P<0.01; ***P<0.001. N0, no regional lymph node metastasis; N1, metastases in 1–3 axillary lymph nodes; N2, metastases in 4–9 axillary lymph nodes; N3, metastases in ≥10 axillary lymph nodes; NOS, not otherwise specified; LBC, lung bronchioloalveolar carcinoma; CCND3, cyclin D3; NCALD, neurocalcin δ; MACF1, microtubule actin crosslinking factor 1; KCTD15, potassium channel tetramerization domain containing 15.

According to the expression profiles included in the GSE113852 and GSE130779 datasets, the expression levels of RAMP2-AS1, CD44, CCND3, NCALD, MACF1 and KCTD15 were significantly downregulated in LUAD compared with in normal tissues (Fig. 2). As shown in Fig. 3, the expression levels of CD44, CCND3, NCALD, MACF1 and KCTD15 were downregulated in the vast majority of LUAD histological subtypes compared with in normal samples; additionally, they were differentially expressed according to tumor stage and nodal metastasis status.

**Figure 4.**
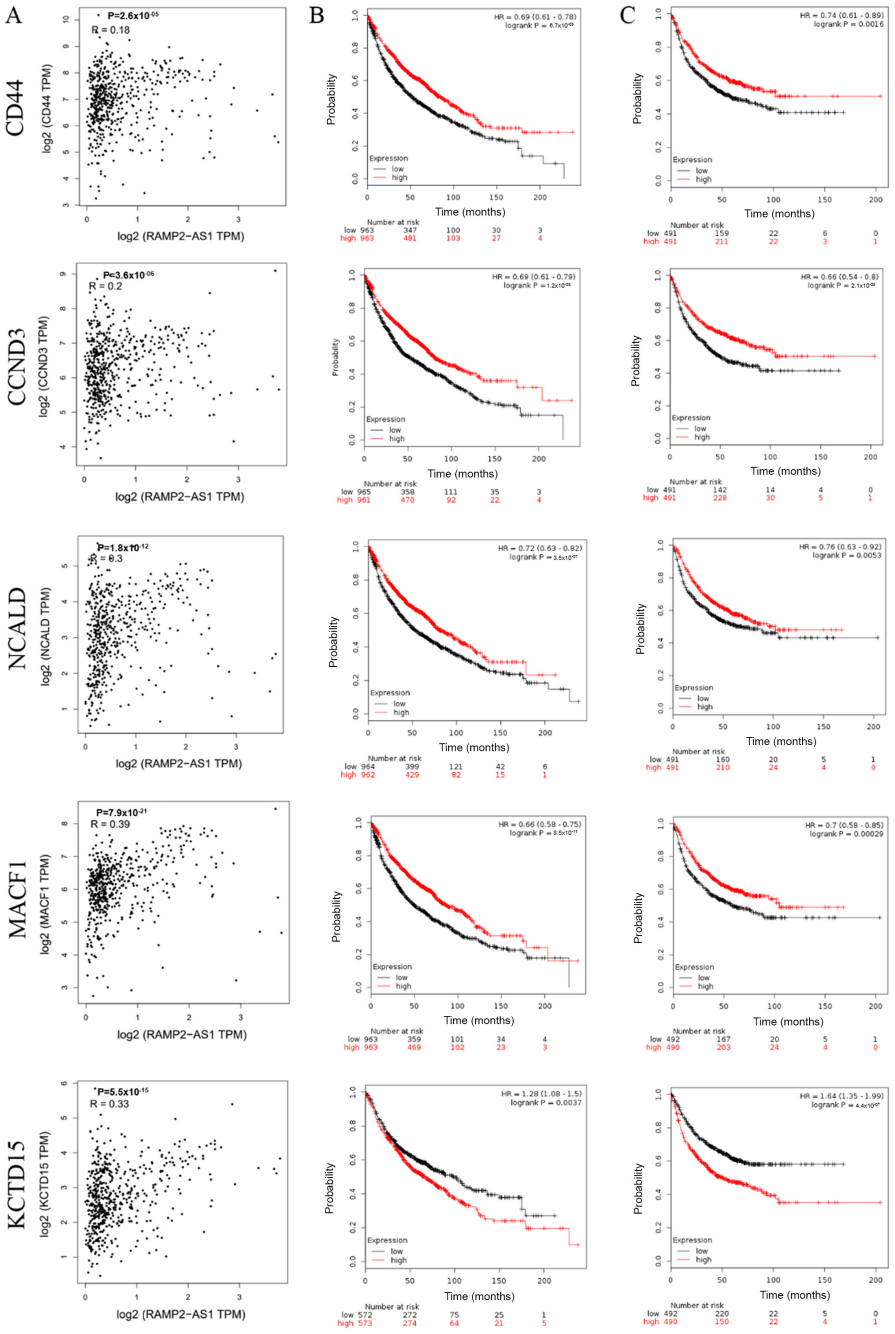
Correlation and prognosis analyses of the selected genes. (A) Correlation analysis of the selected genes as evaluated using Spearman's rank correlation test in Gene Expression Profiling Interactive Analysis. (B) Overall survival and (C) first progression curves of the selected genes in patients with lung adenocarcinoma using Kaplan-Meier plotter analysis. TPM, transcripts per million; HR, hazard ratio; CCND3, cyclin D3; NCALD, neurocalcin δ; MACF1, microtubule actin crosslinking factor 1; KCTD15, potassium channel tetramerization domain containing 15.

The correlation between the expression levels of RAMP2-AS1 and CD44, CCND3, NCALD, MACF1 and KCTD15 in LUAD was evaluated using Pearson's rank correlation test in GEPIA, revealing that RAMP2-AS1 expression was significantly positively correlated with the expression levels of CD44, CCND3, NCALD, MACF1 and KCTD15 (Fig. 4A; P<0.05; R>0). Additionally, the prognostic value of each key mRNA was determined using Kaplan-Meier plotter analysis. The analysis indicated that high expression levels of CD44, CCND3, NCALD and MACF1 resulted in an improved OS and FP in patients with LUAD, while KCTD15 expression exhibited the opposite effect (Fig. 4B and C). Therefore, KCTD15 was excluded from subsequent analyses.

#### Functional enrichment analysis

**Figure 5.**
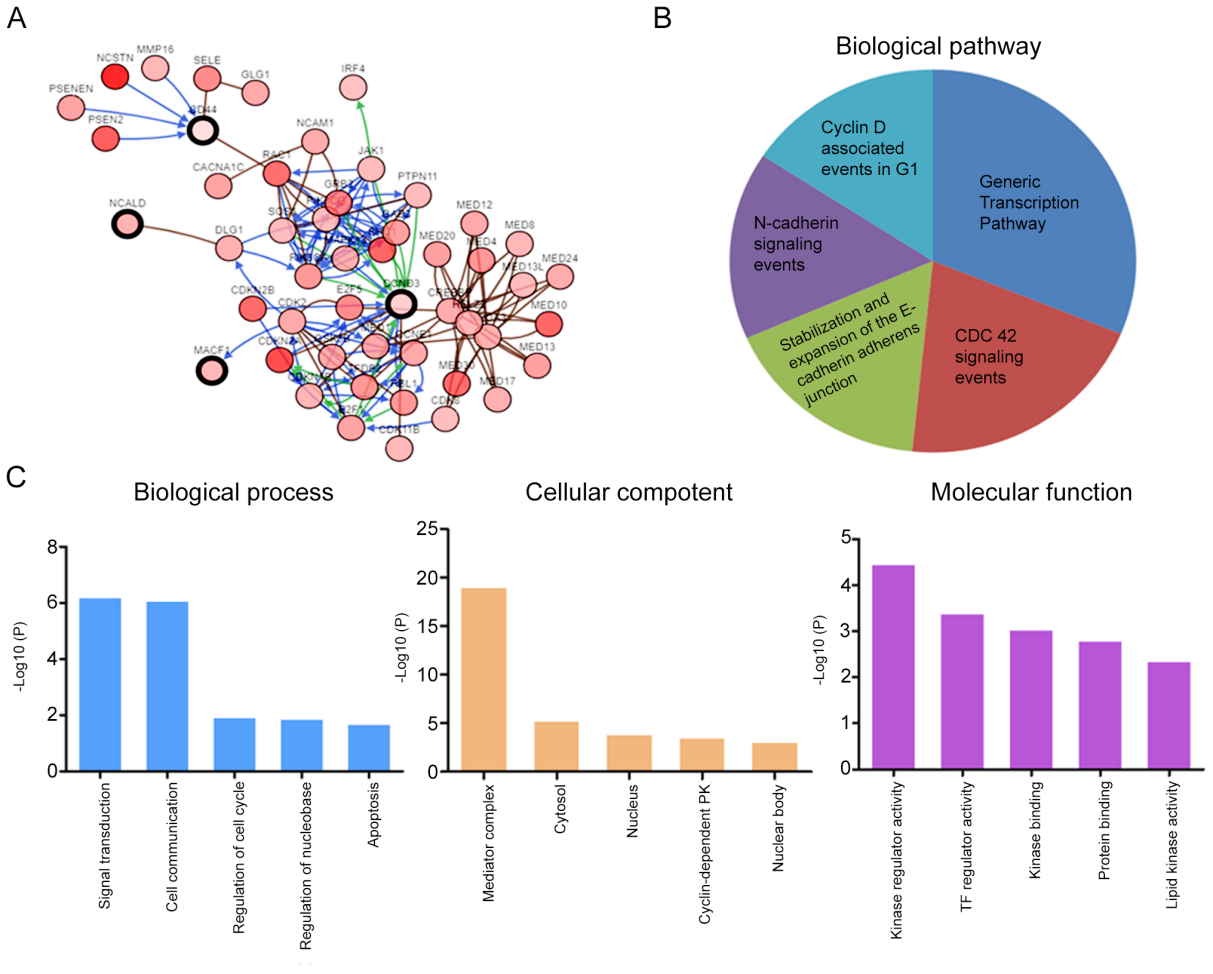
GO and KEGG analyses of the predicted genes. (A) Interactions between the selected genes (n=4) and the predicted ones (n=50). (B) KEGG and (C) GO enrichment analyses of the predicted genes. GO, Gene ontology; KEGG, Kyoto Encyclopedia of Genes and Genomes; CCND3, cyclin D3; NCALD, neurocalcin δ; MACF1, microtubule actin crosslinking factor 1; KCTD15, potassium channel tetramerization domain containing 15.

The co-expression analysis of CD44, CCND3, NCALD and MACF1 was assessed using cBioPortal. The analysis revealed 50 genes that may be associated with CD44, CCND3, NCALD and MACF1 (Fig. 5A). Subsequently, these genes were subjected to GO and KEGG enrichment analyses in FunRich to determine their possible molecular functions. KEGG pathway analysis revealed that these genes were enriched in the ‘cyclin D associated events in G_1_’, ‘generic transcription pathway’, ‘CDC42 signaling events’, ‘stabilization and expansion of the E-cadherin adherens junction’ and ‘N-cadherin signaling events’ (Fig. 5B). The genes involved in the enriched pathways are shown in Table II. Furthermore, GO analysis revealed that the predicted genes were mainly enriched in biological processes such as ‘cell communication’ and ‘signal transduction’, cellular components such as ‘mediator complex’, ‘cytosol’ and ‘nucleus’, and molecular functions such as ‘kinase regulator activity’, ‘TF regulator activity’ and ‘kinase binding’ (Fig. 5C).

#### Construction of interaction networks

**Figure 6.**
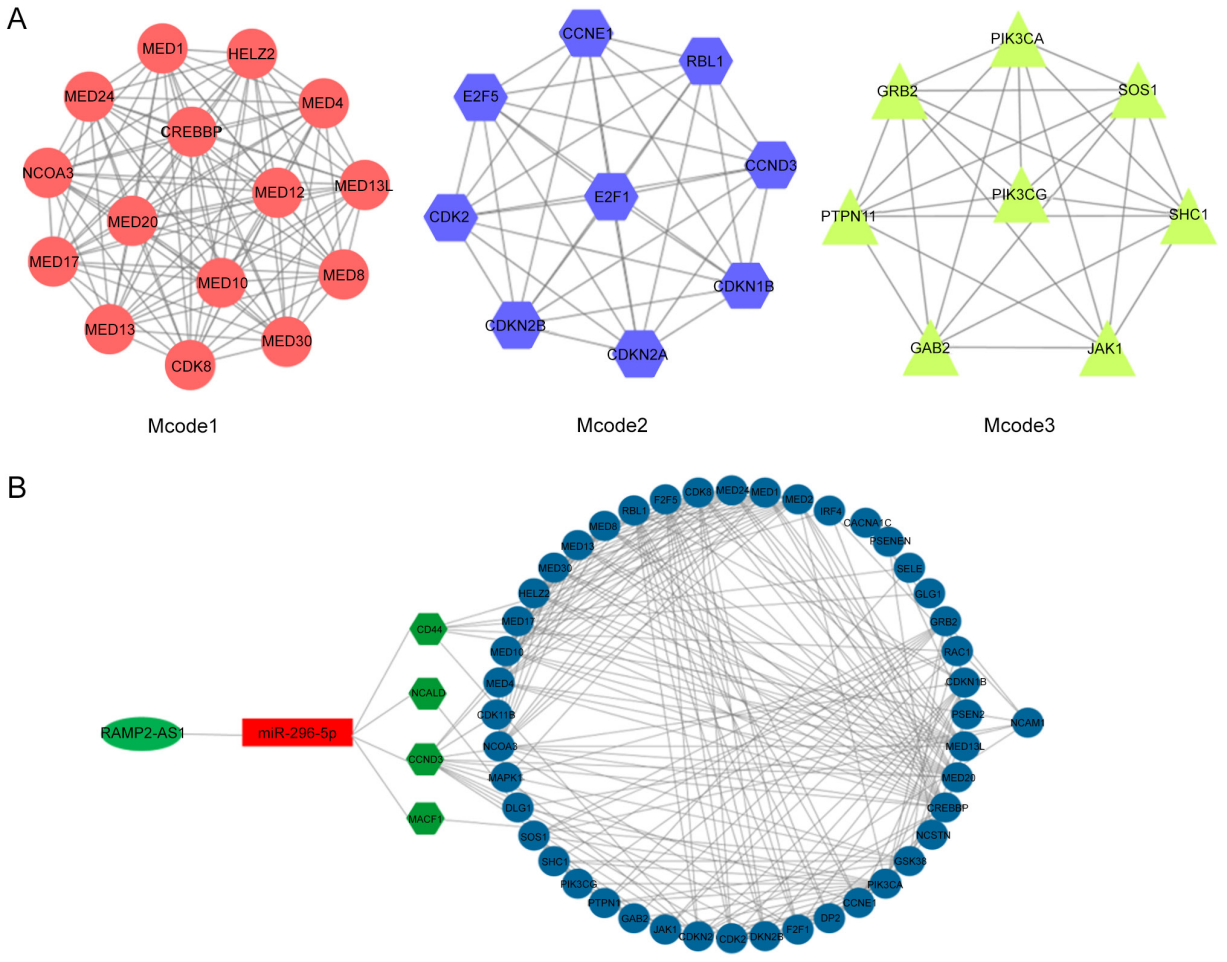
Mcode analysis of the predicted genes and protein-protein interaction network of the selected genes. (A) Network of three enriched modules as analyzed using Cytoscape. (B) Protein-protein interaction network of the selected genes. CCND3, cyclin D3; NCALD, neurocalcin δ; MACF1, microtubule actin crosslinking factor 1; RAMP2-AS1, receptor activity modifying protein 2-antisense RNA 1; miR, microRNA.

Subsequently, the aforementioned 50 genes were subjected to the STRING database analysis to construct the predicted protein-protein interaction network. The interaction network was then imported into the Cytoscape software. Using the Mcode function of Cytoscape, three enriched modules were identified that exhibited a marked overlap with the genes identified in the previous enrichment analysis (Fig. 6A). The functions of these three modules were mainly enriched in these processes, namely ‘generic transcription pathway’, ‘cyclin D associated events in G_1_’, ‘N-cadherin signaling events’, ‘stabilization and expansion of the E-cadherin adherens junction’ and ‘CDC42 signaling events’ (Fig. 5B), reflecting the main functions of the network composed of RAMP2-AS1 and the target genes CD44, CCND3, NCALD and MACF1. Subsequently, Cytoscape was used for the visualization of the protein-protein interaction network of RAMP2-AS1, miR-296-5p, CD44, CCND3, NCALD and MACF1, shown in Fig. 6B.

#### Expression levels of lncRNA RAMP2-AS1, CD44, CCND3, NCALD, MACF1 and miR-296-5p in tumor tissues

**Figure 7.**
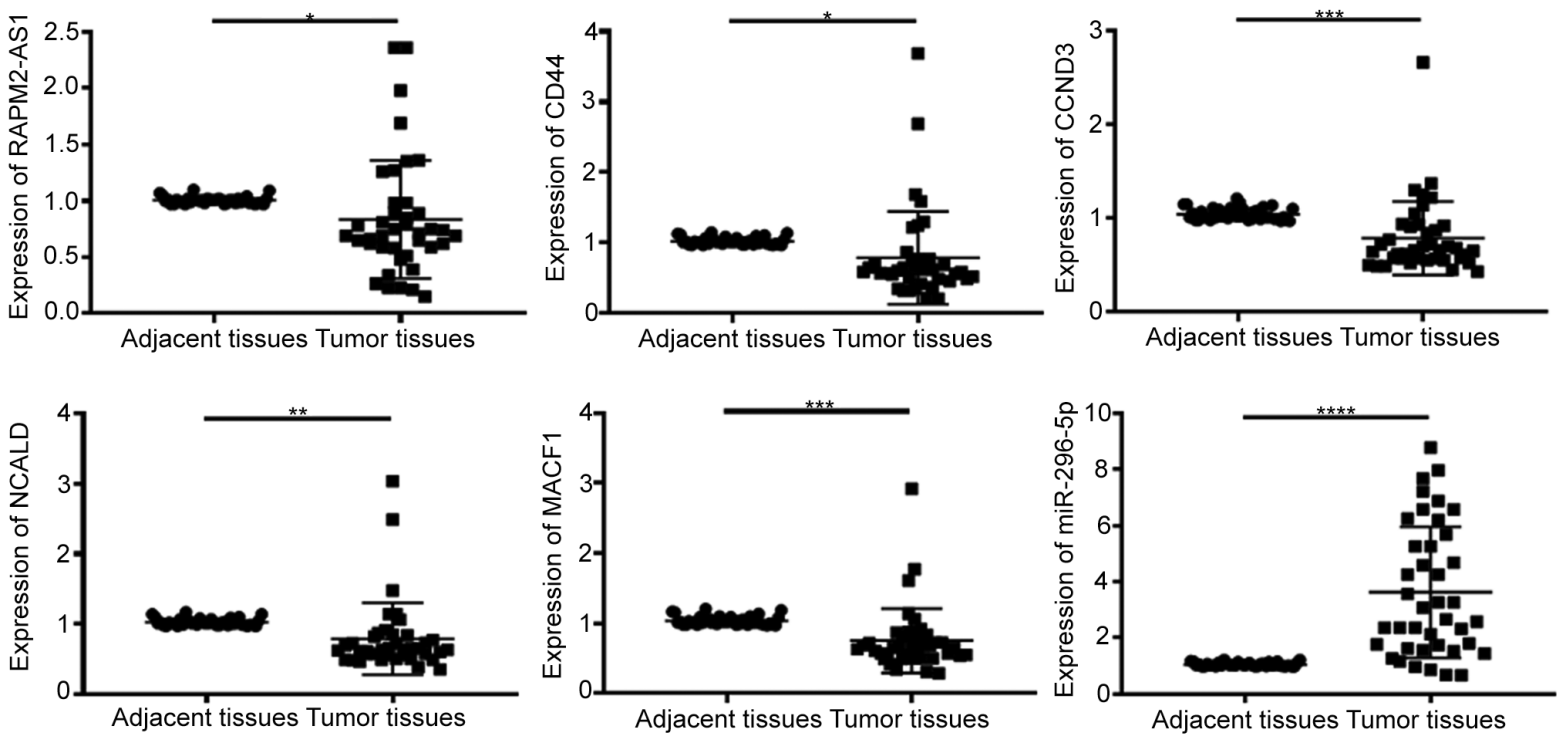
Expression levels of RAMP2-AS1, CD44, CCND3, NCALD, MACF1 and miR-296-5p in tumor and adjacent tissues. lncRNA RAMP2-AS1, CD44, CCND3, NCALD and MACF1 expression was significantly downregulated, while miR-296-5p expression was significantly upregulated in tumor tissues compared with in adjacent normal tissues. *P<0.05; **P<0.01; ***P<0.001; ****P<0.0001. lncRNA RAMP2-AS1, long non-coding RNA receptor activity modifying protein 2-antisense RNA 1; CCND3, cyclin D3; NCALD, neurocalcin δ; MACF1, microtubule actin crosslinking factor 1; miR, microRNA.

The expression levels of RAMP-AS1, miR-296-5p, CD44, CCND3, NCALD and MACF1 were detected in 40 tumor and adjacent tissue samples via RT-qPCR. The results demonstrated that the expression levels of RAMP2-AS1 (P<0.05), CD44 (P<0.05), CCND3 (P<0.001), NCALD (P<0.01) and MACF1 (P<0.001) were significantly downregulated, while miR-296-5p expression (P<0.0001) was significantly upregulated in tumor tissues compared with in adjacent tissues (Fig. 7), which was consistent with the results obtained with the bioinformatics analysis.

#### Correlation analysis

**Figure 8.**
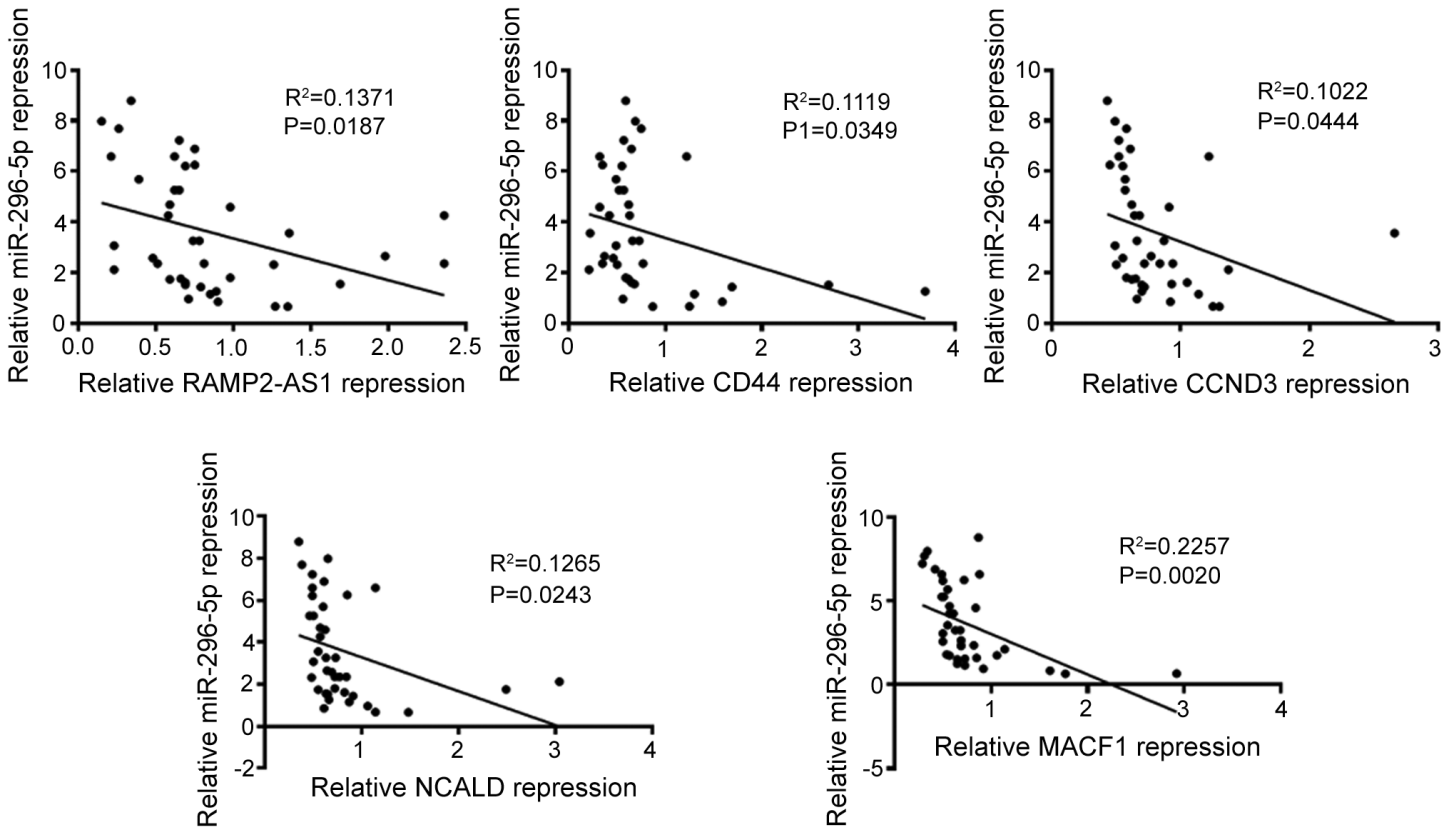
miR-296-5p expression is negatively correlated with the expression levels of RAMP2-AS1, CD44, CCND3, NCALD and MACF1 in tumor tissues. RAMP2-AS1, receptor activity modifying protein 2-antisense RNA 1; CCND3, cyclin D3; NCALD, neurocalcin delta; MACF1, microtubule actin crosslinking factor 1; miR, microRNA.

**Figure 9.**
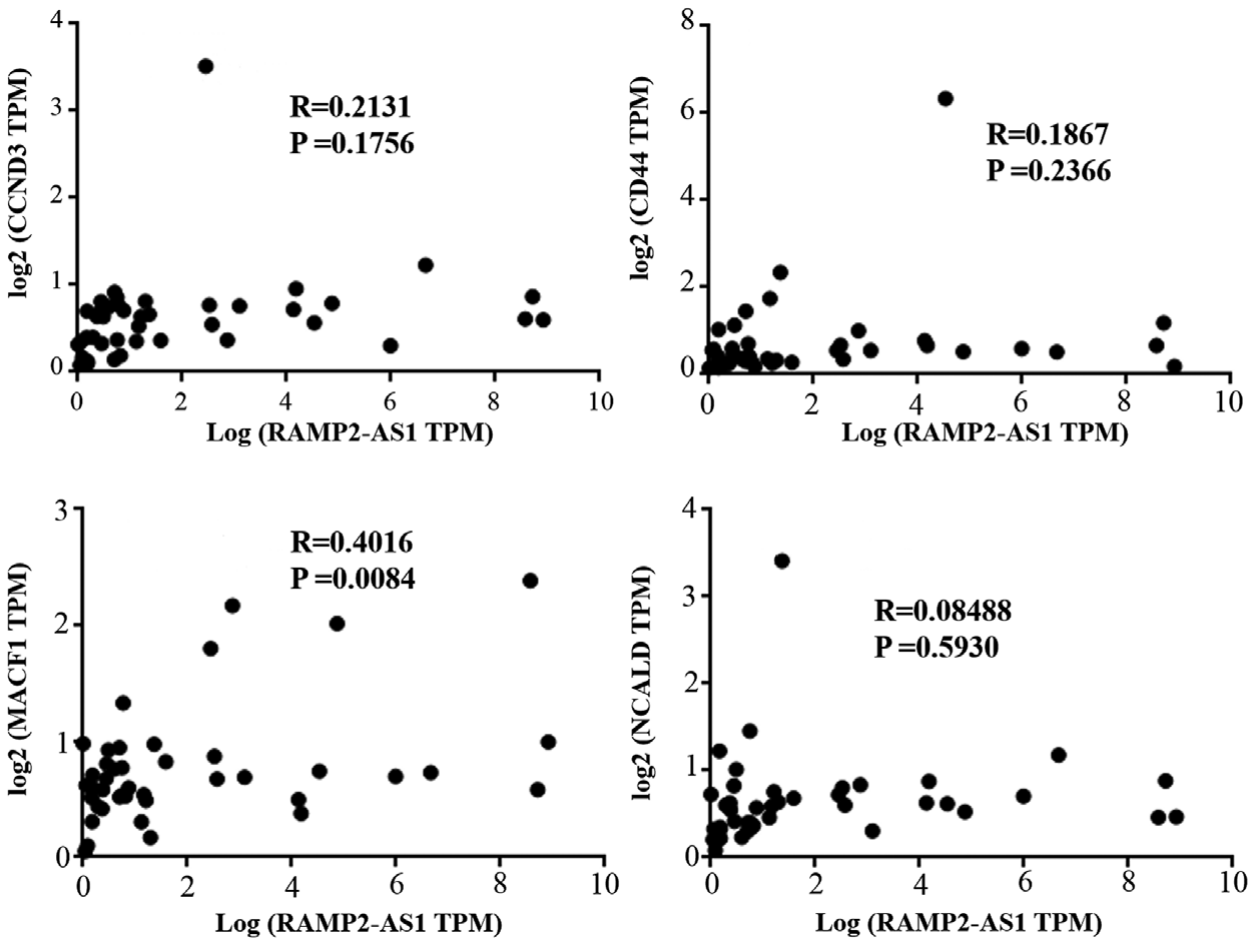
Correlation analysis of the selected genes measured via reverse transcription-quantitative PCR. CCND3, cyclin D3; NCALD, neurocalcin delta; MACF1, microtubule actin crosslinking factor 1; RAMP2-AS1, receptor activity modifying protein 2-antisense RNA 1; TPM, transcripts per million.

In addition, the correlation between the expression levels of miR-296-5p and those of RAMP2-AS1, CD44, CCND3, NCALD and MACF1 was evaluated (Fig. 8). The results revealed that the expression levels of miR-296-5p were negatively correlated with that of RAMP2-AS1 (R^2^=0.1371; P=0.0187), CD44 (R^2^=0.1119; P=0.0349), CCND3 (R^2^=0.1022; P=0.0444), NCALD (R^2^=0.1265; P=0.0243) and MACF1 (R^2^=0.2257; P=0.0020), as analyzed using Spearman's correlation. Subsequently, Pearson's correlation analysis was performed between the expression levels of RAMP2-AS1 and those of CD44, CCND3, NCALD and MACF1 in the tumor tissues of the aforementioned 40 patients. The results revealed that RAMP2-AS1 expression was significantly positively correlated with that of MACF1 (Fig. 9; R=0.4016; P=0.0084), which was consistent with the results obtained with the bioinformatics analysis.

## Discussion

In recent years, the microarray technology has been considered as an effective method to identify differentially expressed genes. An increasing number of studies have demonstrated that dysregulated genes serve a key role in the occurrence and development of LUAD (30–32). In the present study, three differentially expressed lncRNAs and 368 mRNAs were identified from the GSE113852 and GSE130779 datasets. RAMP2-AS1 was selected as a key lncRNA for subsequent analyses, as it was significantly downregulated in LUAD, as well as in most types of cancer, and its upregulation was associated with improved OS. Consistent with these findings, a previous study has demonstrated that RAMP2-AS1 expression is significantly decreased in primary glioblastoma tissues compared with in normal brain tissues and that its decreased expression levels are associated with poor OS in patients with glioblastoma (33). Additionally, RAMP2-AS1 expression in the serum of patients with chondrosarcoma is closely associated with local invasiveness, distant metastasis and poor prognosis in patients with chondrosarcoma (15). Overexpression of RAMP2-AS1 decreases the proliferation of glioblastoma cells *in vitro*, as well as glioblastoma xenografts *in vivo* (33). The aforementioned studies suggest that RAMP2-AS1 may be used as a biomarker for the prognosis of LUAD.

Furthermore, it has been reported that lncRNA RAMP2-AS1 in exosomes derived from chondrosarcoma cells may act as a ceRNA, which combined with miR-2355-5p may modulate VEGFR2 expression, thus positively regulating the angiogenic ability of HUVECs (15). Therefore, the present study hypothesized that RAMP2-AS1 may act as a ceRNA to bind to miRNAs, regulate the expression of target genes and affect the occurrence of LUAD. Therefore, miR-296-5p was identified to bind to RAMP2-AS1 as a ceRNA via the Starbase online database, and high miR-296-5p expression was detected in LUAD. Numerous studies have demonstrated the impact of lncRNA-miRNA-mRNA functional networks on the tumorigenesis of human carcinoma (34–36). For example, it has been revealed that NEAT1 promotes the development of hepatocellular carcinoma cells via regulating the miR-296-5p/CNN2 axis (37). Chen *et al* (38) indicated that lncRNA Forkhead box D3 antisense RNA 1 exerted antitumor effects via upregulating miR-296-5p expression in thyroid cancer. Based on the ceRNA hypothesis, 5 mRNAs (CD44, CCND3, NCALD, MACF1 and KCTD15) were identified as target genes for miR-296-5p. Notably, the expression levels of RAMP2-AS1, CD44, CCND3, NCALD, KCTD15 and MACF1 were all downregulated in LUAD tissues compared with in normal tissues, and RAMP2-AS1 expression was positively correlated with the expression levels of CD44, CCND3, NCALD and MACF1 through database analysis. Additionally, the present study revealed that the expression levels of these 5 mRNAs affected the prognosis of patients with LUAD, suggesting that patients with high expression levels of the 5 mRNAs survived longer than those with low expression levels. The results of the Kaplan-Meier plotter analysis determined that the high expression groups of CD44, CCND3, NCALD and MACF1 had an improved prognosis compared with the low expression groups, while KCTD15 exhibited the opposite trend. To the best of our knowledge, KCTD15 upregulation has never been reported to be associated with pathological states, although it has been indirectly associated with several types of cancer, such as pleomorphic adenoma and medulloblastoma (39,40). The specific role of KCTD15 in LUAD should be investigated in future studies. The present data indicated that RAMP2-AS1 may act as a ceRNA to bind miR-2355-5p, regulate its target genes CD44, CCND3, NCALD and MACF1, and then affect the development of LUAD. Furthermore, the expression levels of CD44, CCND3, NCALD, MACF1 and KCTD15 were downregulated in the vast majority of LUAD histological subtypes compared with in normal samples, and they were differentially expressed according to tumor stage and nodal metastasis status. However, a limitation of the present study is that no comparison was made between these expression levels and patient clinicopathological characteristics, and prognosis data was not collected for the 40 clinical samples.

Screening using the cBioPortal and FunRich tools revealed that 50 genes were closely associated with CD44, CCND3, NCALD and MACF1. These genes were enriched in the ‘cyclin D associated events in G_1_’, ‘generic transcription pathway’, ‘CDC42 signaling events’, ‘stabilization and expansion of the E-cadherin adherens junction’ and ‘N-cadherin signaling events’. In addition, using the STRING database and Cytoscape, three enriched modules among these 50 genes were predicted. The genes involved in the enriched modules overlapped with those identified in the enrichment analysis, thus indicating that ‘generic transcription pathway’, ‘cyclin D associated events in G_1_’, ‘N-cadherin signaling events’, ‘stabilization and expansion of the E-cadherin adherens junction’ and ‘CDC 42 signaling events’ were the main functions of the network composed of RAMP2-AS1, miR-296-5p, CD44, CCND3, NCALD and MACF1. The data further determined that miR-296-5p expression was negatively correlated with that of RAMP2-AS1, CD44, CCND3, NCALD and MACF1, while RAMP2-AS1 expression was positively correlated with MACF1 expression.

MACF1 is a spectraplakin cytoskeletal crosslinking protein that can decrease the toxicity to normal tissues while improving the efficacy of radiation (41). It has been used as a targeted diagnostic marker for glioblastoma (42). Notably, a previous study has suggested that MACF1 mutations are associated with HPV-negative vulvar cancer (43). It is well known that EGFR mutations can influence the prognosis of cancer. EGFR may be used as a promising therapeutic target and EGFR mutations are associated with a poor prognosis in patients with ovarian cancer (44). However, the specific mechanism in LUAD remains unclear. It is well known that miRNAs can target one or more genes to affect their role in tumors. Due to the lack of stratification based on EGFR mutations, the potential association between key genes and EGFR mutations has not been clarified in the current study, which is another limitation that should be further investigated in future research.

In the present study, various databases were used to analyze genes, which may be different from the selected databases of other studies. For example, the two datasets used by Li *et al* (11) consisted of non-metastatic and metastatic samples, while another study directly derived data from TCGA database (12). In addition, the present study performed differential gene expression, functional enrichment, molecular network and prognostic analyses, as well as analyzing the correlation between gene expression levels and different modules, using the GEPIA database to achieve data visualization, which is an advantage of this study. However, *in vitro* experiments to corroborate the results of the present database analysis were not performed, and the current results are not comprehensive. Therefore, further investigations should be performed.

Overall, the present study suggested that miR-296-5p, RAMP2-AS1, CD44, CCND3, NCALD and MACF1 may serve as potential reliable biomarkers for the detection of LUAD, and provided a possible theoretical basis for the pathogenesis of LUAD. However, the possible molecular mechanisms associated with the described ceRNA regulatory network were based on bioinformatics analyses and basic *in vitro* experiments in LUAD tissues. Therefore, the underlying regulatory mechanism should be further investigated in future research.

## Figures and Tables

**Table I.** List of primers used in the present study.

Name	Sequences (5′-3′)
CD44 F	GACAACGCAGCAGAGTAA
CD44 R	TGTGTGGGTAATGAGAGGTA
NCALD F	TCATCGCCTTGAGTGTAA
NCALD R	CCGTCTCTATTGGTGTCC
CCND3 F	CACACCACATCTAAGCCTGAA
CCND3 R	CCCAATCCAAATGCAATAAC
MACF1 F	CTGTGCCTGTGTGTTGAG
MACF1 R	TGGACTGCGTGGTTTTAG
RAMP2-AS1 F	CTTGGATCATGGGCACGGAT
RAMP2-AS1 R	GTCAAGTCACCTCTTGCCCT
GAPDH F	GAAAGCCTGCCGGTGACTAA
GAPDH R	GCATCACCCGGAGGAGAAAT

F, forward; R, reverse; CCND3, cyclin D3; NCALD, neurocalcin δ; MACF1, microtubule actin crosslinking factor 1; RAMP2-AS1, receptor activity modifying protein 2-antisense RNA 1.

**Table II.** Information of long non-coding RNAs in the GSE113852 and GSE130779 datasets.

Name	GSE113852 LogFC	GSE130779 P-value	LogFC	P-value
RAMP2-AS1	−2.65	2.06×10^−18^	−3.16	7.52×10^−3^
ADAMTS9-AS2	−1.66	6.70×10^−16^	−2.42	6.04×10^−4^
Linc00312	−1.58	1.45×10^−11^	−2.97	3.73×10^−4^

FC, fold-change; RAMP2-AS1, receptor activity modifying protein 2-antisense RNA 1.

## Data Availability

All data generated or analyzed during this study are included in this published article. The datasets generated and/or analyzed during the current study are available in the Gene Expression Profiling Interactive Analysis (http://gepia.cancer-pku.cn/detail.php), the cBio Cancer Genomics Portal (cBioPortal; http://cbioportal.org) and the UALCAN platform (http://ualcan.path.uab.edu/).
